# Mobility of Stored Product Beetles after Exposure to a Combination Insecticide Containing Deltamethrin, Methoprene, and a Piperonyl Butoxide Synergist Depends on Species, Concentration, and Exposure Time

**DOI:** 10.3390/insects11030151

**Published:** 2020-02-29

**Authors:** Frank H. Arthur, Christos G. Athanassiou, W. Robert Morrison

**Affiliations:** 1 USDA, Agricultural Research Service, Center for Grain and Animal Health Research, 1515 Manhattan, KS 66502, USA; athanassiou@uth.gr (C.G.A.); william.morrison@usda.gov (W.R.M.III); 2 Department of Agriculture, Crop Production and Rural Environment, University of Thessaly, Phytokou str. N. Ionia, 38843 Volos, Greece

**Keywords:** chemical control, insect growth regulator, sublethal effects, indirect toxicity, movement, postharvest

## Abstract

Adults of *Rhyzopertha dominica* (F.), the lesser grain borer, *Cryptolestes ferrugineus* (Stephens), the rusty grain beetle, and *Sitophilus oryzae* (L.), the rice weevil, were exposed for 1, 24, and 72 h on wheat treated with concentrations of 0% (untreated controls) to 100% of the proposed label rate of an experimental formulation of deltamethrin + Methoprene + piperonyl butoxide synergist. Movement and velocity of movement were assessed after each exposure time using a camera-based monitoring system (Ethovision^®^). Movement of *R. dominica* decreased with increasing concentration and exposure time, so that movement had virtually ceased at the 48 and 72 h exposures. *Cryptolestes ferrugineus* was less susceptible compared to *R. dominica*, but there was still a general pattern of decreased movement and velocity of movement with increasing concentration and exposure time. *Sitophilus oryzae* was the least susceptible species, with less differences at the 1 h exposure interval compared to the other two species, but after 24–72 h, the patterns of declining movement and velocity were apparent as the concentration increased. Data were analyzed using curve-fit equations to show the relationship between concentration and exposure time for each species. Results show that the Ethovison system can be used to assess the sub-lethal effects of exposure to grain protectant insecticides and elucidate behavioral variation between different stored product insects.

## 1. Introduction

The use of the insect growth regulator (IGR) methoprene for direct application to stored grains, and as structural treatments in storage and processing facilities, has been reported in on-going projects for more than 20 years [1,2,3,4,5]. In general, methoprene and IGRs can be reliable alternatives to traditional neurotoxic insecticides including organophosphorous (OP) and pyrethroid compounds [3,6]. However, IGRs generally do not kill adult insects and must be combined with a contact toxicant to target adults. Historical data and recent research show that *Sitophilus oryzae* (L.), the rice weevil, is much more tolerant to pyrethroids used as contact insecticides on stored grains and on treated surfaces, compared to *Rhyzopertha dominica* (F.), the lesser grain borer [7,8]. Nonetheless, recent research shows that a combination of methoprene with the pyrethroid deltamethrin may still give enhanced control of *S. oryzae* [9].

The current commercial formulation of deltamethrin plus methoprene used in the United States (US) is Diacon IGR+^®^ (Central Life Sciences, Schaumberg, IL, USA). This commercial formulation contains 50 mg active ingredient [AI]/mL of deltamethrin and 120 mg [AI]/mL of the IGR methoprene. It can be applied at two label rates, 0.5 ppm deltamethrin + 1.25 ppm methoprene, or 1.0 ppm deltamethrin + 2.5 ppm methoprene. Methoprene alone will control *R. dominica* because the female lays an egg outside the grain kernel, requiring the larva to bore into the kernel upon hatching. Thus, there is a brief time where the larva is vulnerable to the insecticide. Addition of deltamethrin will provide an extra benefit in toxicity to exposed adults. By contrast, *S. oryzae* oviposits directly into a grain kernel, and thus the larva will not be exposed to Methoprene. Hence, different life history traits by stored product species require a contact toxicant to ensure control of adults for species that are able to escape exposure to the IGR.

Methoprene is effective for control of *R. dominica* and external feeders of stored grains [10,11], and for application to surface substrates [2]. Exposure to methoprene may also reduce fecundity of exposed parental adults [1,6,12,13,14]. Inclusion of a neurotoxic contact insecticide could prevent dispersal of exposed adults from treated grain, and potentially limit oviposition elsewhere in the food facility due to immobilization of the adults on the treated grain. Nevertheless, the mobility of adult stored product beetles after exposure to methoprene combined with deltamethrin has not been examined in detail, apart from visual observations confirming knockdown effects. 

Guedes et al. [15] and Morrison et al. [16] used Ethovision^®^, a system that is designed to continuously monitor movement of an individual insect on a target area or surface. The time to knockdown of adults, and immobilization patterns during or after exposure, have been used as indicators of insecticidal efficacy and the presence of phosphine resistance [17,18]. Exposure to non-neurotoxic insecticides, such as the pyrrole chlorfenapyr, resulted in reduced total movement after exposure, along with increased erratic movement as measured by angular velocity [14]. Understanding the range of indirect toxicity effects on the behavior of insects may help to provide food facility managers with a more comprehensive assessment of the efficacy by chemical control measures [15]. Furthermore, the effects on mobility may be used to predict mortality and concomitant efficacy [15,16].

Based on the results above, further elucidating mobility after insecticidal exposure may be used to develop a comprehensive understanding of insecticide efficacy, and predict eventual mortality, as initial changes in mobility patterns may be correlated with mortality [18,19]. Thus, the objectives of this study were to (1) develop an experimental methodology for using Ethovision^®^ to examine the mobility of stored product beetles after exposure to an insecticide, and (2) to evaluate movement after exposure to an insecticide that is under development that includes deltamethrin, methoprene, along with the synergist, piperonyl butoxide. The three beetle species used in this study were adults of *R. dominica*, *S oryzae*, and *Cryptolestes ferrugineus* (Stephens), the rusty grain beetle.

## 2. Materials and Methods

### 2.1. Insects and Commodity

All insect cultures of the species listed above were maintained at the USDA-ARS-Center for Grain and Animal Health Research (CGAHR), Manhattan, KS, USA. All species had been maintained at the CGAHR for more than 30 years. *Rhyzopertha dominica* and *S. oryzae* were reared on whole kernels of mixed-variety hard red winter wheat kernels, while *C. ferrugineus* was reared on a diet of rolled oats. All species were reared inside Percival incubators (Percival, Perry, IA, USA), at 27 °C and 65% relative humidity (RH), in total darkness. The adults of all species used in the test were less than 3 weeks old. 

### 2.2. Insecticide Assay

The amount of wheat treated for each replicate was 500 g. The insecticide used in the test was an experimental formulation containing 12 mg AI/mL of deltamethrin, 27 mg AI/mL methoprene, and 320 mg AI/mL piperonyl butoxide (PBO) synergist. The proposed application rate for this experimental formulation was 1138 mL in 18,920 mL water per 27,272.7 kg of wheat. The equivalent volume rate for 500 g is 0.35 mL. This amount of formulation needed 0.042 mL, which was too small to formulate in 0.35 mL, so 1.5 mL of formulation was mixed in 25 mL of water, to apply at 0.35 mL/500 g. This represented the maximum concentration, which was 100% of the proposed label rate. The other application rates used for the study were 75, 50, 25, and 10% of the proposed rate. This required mixing 1.13 mL of the parent formulation in 25 mL water for the 75% rate, 0.75 mL in 25 mL water for the 50% rate, 0.375 mL in 50 mL water for the 25% rate, and 0.150 mL in 50 mL water for the 10% rate. 

Each concentration was replicated three separate times as blocks. For the first replicate, the 500 g of wheat for each concentration was spread out over a flat surface, and an artist’s air brush (Badger 100, Badger Corporation, Franklin Park, IL, USA) was used to mist the solution evenly, and directly onto the wheat. An untreated control (0% concentration) consisted of using distilled water. After a replicate and concentration was treated, the wheat was subdivided into three 120 mL vials containing 100 g of treated wheat each. 

Tests were done with each species in succession. First, 50–60 *R. dominica* were introduced into one of the vials from each replicate and concentration. After insecticide exposure, five adults classified as alive or affected (e.g., exhibiting some level of mobility) were removed from each of the three replicate vials. One adult from each concentration was placed individually into six filter paper-lined (Whatman #1, GE Healthcare, Chicago, IL, USA) 62 cm^2^ Petri dishes, where movement (distance moved and instantaneous velocity) was recorded using a video-tracking apparatus combined with Ethovision XT v. 14.0 (Noldus Software, Leesburg, VA, USA). The apparatus was described in detail in Morrison et al. [16] Briefly, a network camera (Basler AG, Ahrensburg, Germany) suspended 80 cm above the insects recorded the movement of each adult for 10 min, after which the dishes were removed, and the insects were discarded. This process was repeated four more times per replicate, with a total of n = 15 insects tested per treatment combination. After the process was completed for *R. dominica*, 50–60 adults of *S. oryzae* and *C. ferrugineus* were placed on the second and third vial containing 100 g of treated wheat, and the entire process described above was repeated for these species. Mobility was assessed after exposure to the insecticide for 1, 24, and 72 h. No adults of any species were observed flying or crawling under the filter paper while they were observed under the Ethovision system during the 10-min exposures.

### 2.3. Statistical Analysis

Data were analyzed using the Mixed Procedure of the Statistical Analysis System (SAS version 9.2, SAS Institute, Cary, NC, USA). Since different vials were used for different exposure intervals, the data were analyzed following a two-way ANOVA with treatment (100, 75, 50, 25, and 0% of the proposed labeled rate) and exposure time (1, 24, 72 h) as main effects. Distance moved (cm) and instantaneous velocity (cm/min) were the response variables, analyzed separately for each species. Additional analysis on the data was done using the Regression Procedure of the SAS (version 9.4, SAS Institute, Cary, NC, USA). Due to the fact that data were an ordered sequence ranging from 0% (untreated controls) to 100% of the tested concentration, mean separation tests were not done on the data. Instead, a curve-fitting approach was used by processing the data for each species and exposure interval through the Table Curve software package (Version 5.01, Systat, Richmond, CA, USA). This software calculates the maximum R^2^ of any equation that could be fit to the data, along with the R^2^ value of a selected equation. The equations used were simple linear or non-linear equations. The equations were selected from the list provided by the Table Curve software according to their R^2^ value to ensure the relationship to the data was accurately modelled.

## 3. Results

### 3.1. Initial Analysis

Species, exposure time, and concentration were significant at *p* < 0.001 for main effects total distance moved and the instantaneous velocity (Table 1). Species by the three-way interaction was significant (*p* < 0.05). Data were thus analyzed by species and exposure time using the Table Curve software, to select regression equations with concentration as the independent variable and movement and velocity as the dependent variables.

### 3.2. Rhyzopertha Dominica

**Figure 1:**
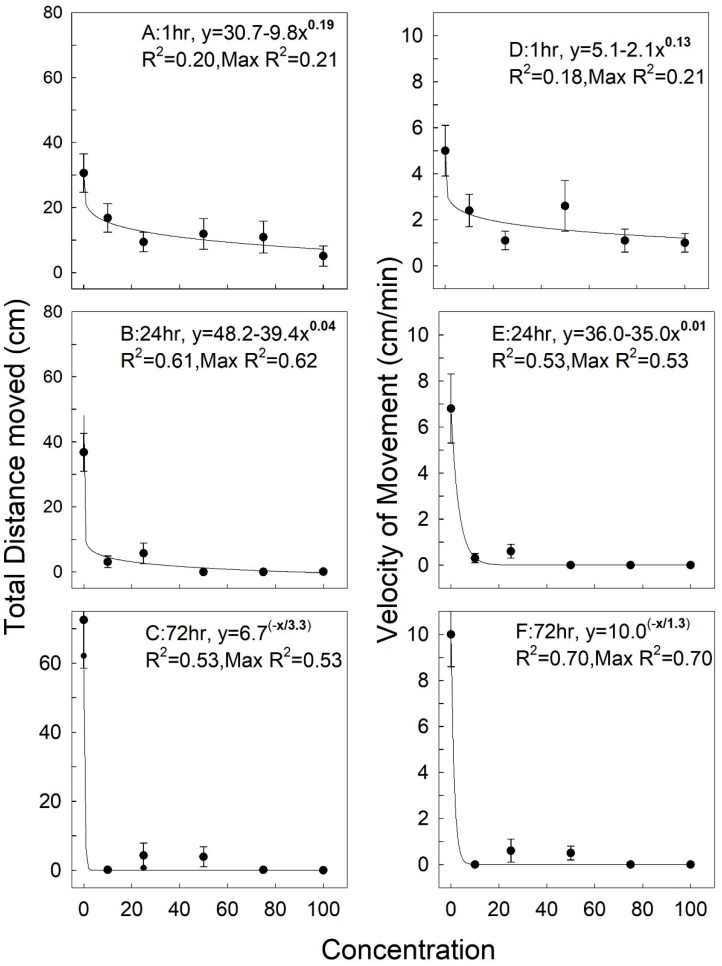
Distance moved (cm) for *R. dominica* exposed for 1, 24, and 72 h on wheat treated with a combination of deltamethrin, methoprene, and piperonyl butoxide (Figure 1A–C), and instantaneous velocity of movement (cm/min) (Figure 1D–F). Concentration ranged from 0% to 100% of an experimental formulation applied at the rate of 12 mg AI/mL of deltamethrin, 27 mg AI/mL methoprene, and 320 mg AI/mL piperonyl butoxide (PBO) synergist (see Materials and Methods). Equations are curve-fit non-linear equations from Table Curve software, and equations were fit to the raw data (n = 15 replicate for each concentration and each exposure time). The R^2^ of the equation is given, along with the maximum R^2^ of any curve that could be fit to the data set.

The relationship between total distance moved, and velocity of movement, for *R. dominica*, with respect to concentration of deltamethrin + methoprene + piperonyl butoxide (PBO), could be described by non-linear equations. These equations were fit to the raw data (but means and standard errors are shown in Figure 1A). After one hour of exposure, the movement of *R. dominica* decreased as the concentration increased from untreated controls (0%) to 100% of the proposed labeled rate (Figure 1A). Total distance moved ranged from 0 to about 68 cm, with considerable variation in the data so that the maximum R^2^ of any equation that could be fit to the data was only 0.21. However, after the 24-h exposures there was little movement of *R. dominica* at concentrations of 50% or above, with less variation in the data (Figure 1B). There was some scattered movement at the 25% and 50% concentrations after the 72-h exposures, with more variation compared to the 24-h exposures (Figure 1C). Velocity of movement paralleled the data for total movement. Velocity ranged from 0 to almost 9 cm/min at the 1-h exposures, with considerable variation, with a general trend of decreasing velocity with increasing concentration (Figure 1D). There was no movement and hence no velocity of movement beyond the 25% concentration at the 24-h exposures (Figure 1E), but there was some movement and associated velocity at the 25 and 50% concentrations at the 72-h exposures (Figure 1F). 

The minimum and maximum vales for movement and velocity are shown in Table 2. There was considerable variation in the data for both treatments and controls at the 1-h exposure interval (Table 2). However, at the 24- and 72-h exposure intervals, movement and velocity both decreased, with less variation at the individual concentrations. It was clear that *R. dominica* was susceptible to the combination insecticide, as reflected in the data for decreased movement and velocity after exposure to the increasing concentrations of the insecticide.

### 3.3. Cryptolestes Ferrugineus

**Figure 2:**
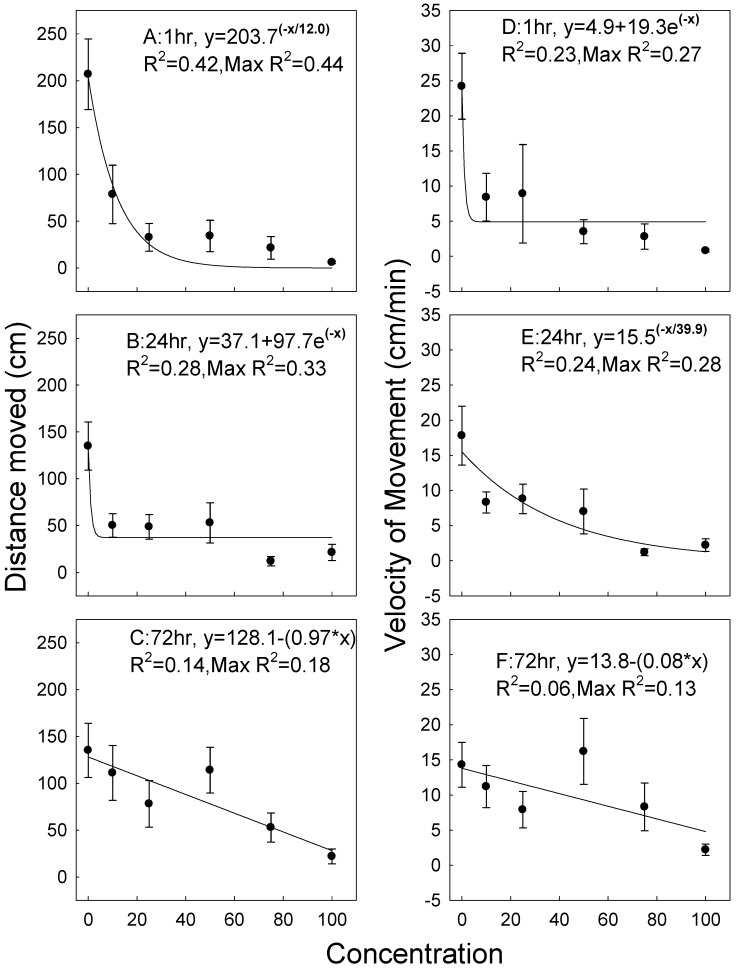
Distance moved for *C. ferrugineus* exposed for 1, 24, and 72 h on wheat treated with the experimental formulation (Figure 2A–C), and velocity of movement exposed at the same times (Figure 2D–F). Curve-fit lines are as described for Figure 1.

This species was much more mobile compared to *R. dominica*, and less susceptible to the insecticide (Figure 2). Total distance moved after the 1-h exposures ranged from 0 to over 400 cm, with decreasing movement, as concentration increased but with variation in the data and movement at all concentrations (Figure 2A). Results were similar at the 24 and 72-h exposures, with a general decline in movement with increasing concentration, but with even more variation and movement at all exposure concentrations (Figure 2B,C). The linear equation for the 72-h data was significant but with a poor fit (Figure 2C). Relative to untreated controls, there was a 60–80% decrease in movement at the 1- and 24-h exposures. At the 72-h exposure, higher concentrations resulted in progressively less movement. Data for velocity of movement also varied considerably at both the 1-h and 24-h exposures, and hence the non-linear equations gave a good fit compared to the maximum R^2^ of any equation that could be fit to the data (Figure 2D,E). Only a linear equation could be fit to the data for the 72-h exposures (Figure 2F). There was also considerable variation in the data for untreated controls, similar to results for *R. dominica*. *Cryptolestes ferrugineus* appeared to be less susceptible to the insecticide compared to *R. dominica*, even though *C. ferrigineus* was clearly the more mobile species. Again, the variation in movement and velocity among the individual concentrations, as shown in the data for minimum and maximum values (Table 3), contributed to the poor fit of the non-linear equations.

### 3.4. Sitophilus Oryzae

**Figure 3:**
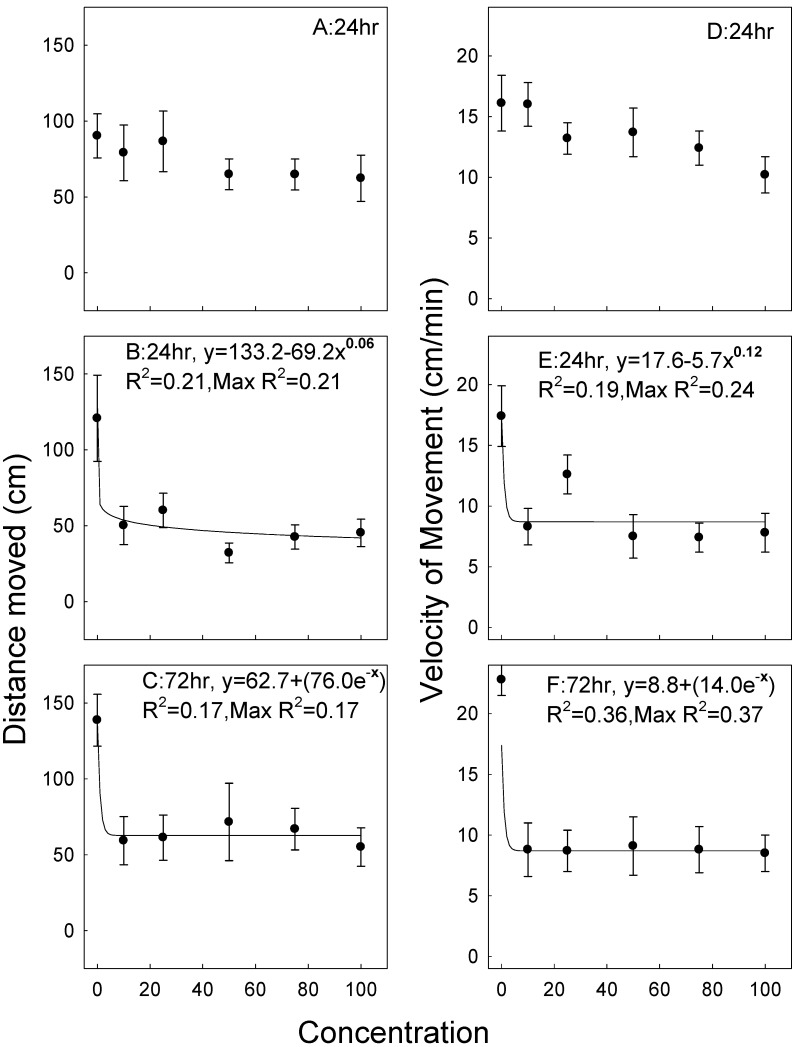
Distance moved for *S. oryzae* exposed for 1, 24, and 72 h on wheat treated with the experimental formulation (Figure 3A–C), and velocity of movement exposed at the same times (Figure 3D–F). Curve-fit lines are as described for Figure 1.

There was considerable movement after exposure to all concentrations and data were so variable after the one-hour exposures that no equations could be fit to the data (Figure 3A). Movement decreased somewhat after the 24-h exposures, with a general decline with increasing concentrations, but again data were so variable that the maximum R^2^ of any equation that could be fit to the data was only 0.21 (Figure 3B). Data were even more skewed at the 72-h exposures, with considerable movement after exposure to all tested concentrations (Figure 3C). However, relative to controls, there was still a 60%–70% decrease (at 24 and 72 h) in the distance moved between 20%–100% the application rate. Data for velocity of movement followed the same trends after the 1-, 24-, and 72-h exposures as the data for total distance moved (Figure 3D–F, respectively). There was also a decrease in velocity as the exposure interval and concentration increased. Data for untreated controls and treatments varied within the individual concentrations, as shown by the minimum and maximum values similar to results for the other two species (Table 4). 

## 4. Discussion

We examined movement patterns of three stored product beetle species, after exposure to a new insecticide for which there are no data available regarding sub-lethal effects. Based on our results, exposure to the new insecticide affected movement and velocity of movement of all three species, though the effect was most pronounced in *R. dominica*. In addition, we did see some indication of increased movement at the 72-h exposures compared to the 24-hr exposures, which was surprising, because in most studies knockdown after exposure usually leads to mortality rather than to recovery [20]. In earlier studies, Arthur [21,22] found that knockdown may lead to recovery in the presence of food sources that are reachable by insects, underlying the importance of sanitation in storage and processing facilities, which can adversely affect chemical control procedures [23]. In our experiments, the exposed adults remained on the commodity, which can be considered a food source. It is unclear if this movement change that we observed during our tests may lead to complete recovery, or will eventually affect parental functionality and progeny production capacity in the treated substrate. Additional experimental research is needed to clarify these effects. From the data available so far, it seems that this effect is realistic for pyrethroids [3], but not in IGRs, where effects on immature life stages are more irreversible [4,10,23]. 

Nonetheless, it is clear that there were significant and sustained sublethal reductions in movement for each species tested, though the strength and timing of onset of the reductions in movement depended on species. In the case of *R. dominica*, sublethal effects manifested after just 1 h of exposure to 20%–100% of the proposed labeled rate, with a corresponding decrease in movement by about two-thirds compared to controls. By contrast, substantial sublethal effects for *C. ferrugineus* and *S. oryzae* did not manifest until 24 h to 72 h after exposure to 20%–100% of the proposed labeled rate. However, for each species, there were substantial reductions in movement even at exposure to low concentrations of the insecticide. This suggests that dispersal from the treated area will be minimal, and these individuals will likely not be able to colonize new areas of a food facility (e.g., including perhaps adjacent grain bins), as has been documented by exposure of *T. castaneum* and *R. dominica* to a long-lasting, deltamethrin-incorporated netting material [16]. Understanding movement after exposure provides a more comprehensive picture of the efficacy for chemical control tactics, with clear management implications. 

The occurrence of knockdown as a desirable characteristic has been extensively debated for decades among researchers. Theoretically, if insects are knocked down quickly, then the uptake of the toxic agent is terminated, and the exposed individuals may detoxify the insecticide and recover [20], which may happen when adult stored product beetles are exposed to pyrethroids, including deltamethrin [3,21]. Conversely, absence of knockdown may allow insects to move away from the treated area, thus avoiding the continuous exposure with this area [3], which may happen to non-neurotoxic AIs, such as chlorfenapyr. Our data show a temporary effect in adult movement, which may be desirable, as the inclusion of a neurotoxic agent along with methoprene slows down the exposed adults without terminating the exposure. As methoprene acts on the immatures, the inclusion of an adulticide, such as deltamethrin, is expected to cause adult mortality [24,25,26,27]. For instance, Athanassiou et al. [28] used a combination of methoprene with natural pyrethrum, and found that all the exposed psocid individuals were dead in a very short interval, an effect that was apparently due to the presence of natural pyrethrum and not to methoprene.

This is the first reported experiment using the Ethovision^®^ system and associated software to assess movement after adult stored product beetles were exposed on a treated grain. Previous tests with this system utilized a treated concrete surface [15,16,29]. In our current test the excessive variation among exposed individuals, and also among untreated controls, presented issues with the analyses that were not expected, given results from the previous studies with treated surfaces. It may be that more replication is needed for studies with treated grains, or more diligence in selecting individuals of similar condition to minimize variation. However, our data presented in this paper does show clear trends in terms of distance and velocity of the exposed adults, even with the variation, namely substantial and often sustained sublethal reductions in movement. Ultimately, we believe that the variation in extremes of movement (e.g., minimums and maximums) was primarily due to the difficulty of objectively quantifying the level of knockdown or incapacitation after exposure to the insecticide. This was mostly attributed to variations in movement characteristics among the insect individuals tested, rather than to actual variations among treatments. Furthermore, the decrease in the values of mobility and velocity were often proportional to the concentrations tested, suggesting that there was a treatment effect. Considering the differences among the species tested, and apart from differences in mobility, which were mostly related with each species, *C. ferrugineus*, the mobile species, was less susceptible than the more sessile *R. dominica*. In summary, the results of the present study show that exposure to the new insecticide does change movement patterns of adult stored product beetles, but this effect will vary according to species, concentration, and exposure interval. The combination of methoprene with other insecticides that can be used against adults may increase the efficacy of the application, as compared with the application of methoprene alone, directly, through increased adult mortality, and indirectly, through reduced movement, oviposition, and other biological processes. 

## 5. Conclusions

The results of this study show that the Ethovision system can be used to assess movement of adult stored product beetles after exposure to an insecticide, and thus provide an estimate of toxicity and susceptibility. Generally, there were substantial sublethal reductions in movement by each species, even after exposure to low concentrations of the insecticide. However, onset and magnitude of these indirect effects will be dependent specifically on exposure interval, insect species, and concentration of the insecticide to which those insects are exposed.

## Figures and Tables

**Table 1:** ANOVA (Proc Mixed, SAS) for main effects species (*R. dominica*, *C. ferrugineus*, *S. oryzae*), exposure time (1, 24, or 72 h, Exposure Time (ET), and concentration of the experimental formulation (Conc.) consisting of methoprene, deltamethrin, and a piperonyl butoxide synergist. Concentrations were 10%, 25%, 50%, 75%, and 100% of the application rate of 1138 mL of the formulation per 27,273 kg of wheat (see description for dilution of concentration in Materials and Methods). Response variables were distance moved (cm) and velocity (cm/min).

Response	Factor	df	F	*p*
Distance Moved	Species	2707	92.5	<0.001
	ET	2707	7.4	<0.001
	Conc.	5707	37.8	<0.001
	Species*ET	4707	1.6	0.162
	Species*Conc.	10,707	5.1	<0.001
	ET*Conc.	10,707	0.7	0.705
	Species*ET*Conc.	20,707	2.4	<0.001
Velocity	Species	2707	25.0	<0.001
	ET	2707	7.4	<0.001
	Conc.	2707	37.8	0.039
	Species*ET	5707	1.6	0.119
	Species*Conc.	4707	5.1	<0.001
	ET*Conc.	10,707	0.7	0.541
	Species*ET*Conc.	10,707	2.4	<0.001

**Table 2:** Minimum (Min) and maximum (Max) values for total distance moved (in cm) and instantaneous velocity (velocity, cm/min) for *R. dominica* during 10 min trials after 1, 24, and 72 h of exposure to the combination treatment of deltamethrin + methoprene + piperonyl butoxide synergist, with a total of n = 15 individuals tested per treatment combination.

Exposure Time	Concentration	Distance Moved		Velocity	
		Min	Max	Min	Max
1 H	0	0.0	66.6	0.0	15.8
	10	0.0	41.7	0.0	8.5
	25	0.0	42.2	0.0	4.7
	50	0.0	48.4	0.0	11.9
	75	0.0	49.8	0.0	5.2
	100	0.0	37.2	0.0	3.7
24 H	0	5.1	66.9	0.5	18.2
	10	0.0	24.7	0.0	2.5
	25	0.0	40.9	0.0	4.1
	50	0.0	0.0	0.0	0.0
	75	0.0	0.0	0.0	0.0
	100	0.0	0.5	0.1	0.0
72 H	0	0.0	218.4	0.0	22.8
	10	0.0	0.5	0.0	0.1
	25	0.0	53.4	0.0	7.1
	50	0.0	30.7	0.0	4.0
	75	0.0	1.0	0.0	0.1
	100	0.0	0.0	0.0	0.0

**Table 3:** Minimum (Min) and maximum (Max) values for total distance moved (in cm) and instantaneous velocity (Velocity, cm/min) for *C. ferrugineus* during 10 min trials after 1, 24, and 72 h of exposure to the combination treatment of deltamethrin + methoprene + piperonyl butoxide synergist, with a total of n=15 individuals tested per treatment combination.

Exposure Time	Concentration	Distance Moved		Velocity	
		Min	Max	Min	Max
1 H	0	0.0	416.6	0.0	55.6
	10	0.0	318.4	0.0	34.3
	25	0.0	179.0	0.0	85.1
	50	0.0	169.2	0.0	17.2
	75	0.0	151.4	0.0	3.4
	100	0.0	18.5	0.0	3.2
24 H	0	9.1	420.5	0.9	59.2
	10	0.5	188.7	0.1	18.9
	25	0.0	154.3	0.0	19.2
	50	0.0	190.9	0.0	33.1
	75	0.0	55.0	0.0	5.5
	100	0.0	105.4	0.1	11.3
72 H	0	4.1	314.6	0.4	37.3
	10	5.2	335.8	0.0	34.1
	25	0.5	242.3	0.0	25.7
	50	6.2	240.7	0.0	65.5
	75	0.0	158.3	0.0	50.2
	100	0.0	97.0	0.0	9.7

**Table 4:** Minimum (Min) and maximum (Max) values for total distance moved (in cm) and instantaneous velocity (Velocity, cm/min) for *S. oryzae* during 10 min trials after 1, 24, and 72 h of exposure to the combination treatment of deltamethrin + methoprene + piperonyl butoxide synergist, with a total of n = 15 individuals tested per treatment combination.

Exposure Time	Concentration	Distance Moved		Velocity	
		Min	Max	Min	Max
1 H	0	13.5	283.0	5.5	29.2
	10	17.6	198.4	6.7	30.8
	25	12.1	205.8	4.9	22.2
	50	8.1	259.2	1.9	25.9
	75	15.1	169.9	6.2	24.9
	100	11.6	191.9	2.9	19.9
24 H	0	0.0	339.0	0.9	59.2
	10	0.5	188.7	0.1	18.9
	25	6.6	180.5	0.0	19.2
	50	0.0	8 7.4	0.0	33.1
	75	0.5	88.8	0.0	5.5
	100	0.0	103.2	0.1	11.3
72 H	0	52.4	272.2	12.4	30.2
	10	0.5	233.8	0.7	25.7
	25	0.0	227.0	0.0	22.7
	50	0.0	258.1	0.0	25.8
	75	0.0	147.7	0.0	22.0
	100	0.0	174.7	0.0	17.5
